# Microfluidic compartmentalization of rat vagal afferent neurons to model gut-brain axis

**DOI:** 10.1186/s42234-023-00140-3

**Published:** 2024-02-21

**Authors:** Gregory Girardi, Danielle Zumpano, Helen Raybould, Erkin Seker

**Affiliations:** 1 Department of Biomedical Engineering, University of California – Davis, Davis, CA 95616 USA; 2 Department of Anatomy, Physiology, and Cell Biology, University of California – Davis, Davis, CA 95616 USA; 3 Department of Electrical and Computer Engineering, University of California – Davis, Davis, CA 95616 USA

**Keywords:** Primary vagal afferent neuron culture, Retrograde transport, Gut-brain axis, Organ-on-a-chip, Microfluidics, microphysiological systems

## Abstract

**Background:**

Vagal afferent neurons represent the key neurosensory branch of the gut-brain axis, which describes the bidirectional communication between the gastrointestinal system and the brain. These neurons are important for detecting and relaying sensory information from the periphery to the central nervous system to modulate feeding behavior, metabolism, and inflammation. Confounding variables complicate the process of isolating the role of the vagal afferents in mediating these physiological processes. Therefore, we developed a microfluidic model of the sensory branch of the gut-brain axis. We show that this microfluidic model successfully compartmentalizes the cell body and neurite terminals of the neurons, thereby simulates the anatomical layout of these neurons to more accurately study physiologically-relevant processes.

**Methods:**

We implemented a primary rat vagal afferent neuron culture into a microfluidic platform consisting of two concentric chambers interconnected with radial microchannels. The microfluidic platform separated cell bodies from neurite terminals of vagal afferent neurons. We then introduced physiologically-relevant gastrointestinal effector molecules at the nerve terminals and assessed their retrograde transport along the neurite or capacity to elicit an electrophysiological response using live cell calcium imaging.

**Results:**

The angle of microchannel outlets dictated the probability of neurites growing into a chamber versus tracking along chamber walls. When the neurite terminals were exposed to fluorescently-labeled cholera toxin subunit B, the proteins were taken up and retrogradely transported along the neurites over the course of 24 h. Additionally, mechanical perturbation (e.g., rinsing) of the neurite terminals significantly increased intracellular calcium concentration in the distal soma. Finally, membrane-displayed receptor for capsaicin was expressed and trafficked along newly projected neurites, as revealed by confocal microscopy.

**Conclusions:**

In this work, we developed a microfluidic device that can recapitulate the anatomical layout of vagal afferent neurons in vitro. We demonstrated two physiologically-relevant applications of the platforms: retrograde transport and electrophysiological response. We expect this tool to enable controlled studies on the role of vagal afferent neurons in the gut-brain axis.

**Supplementary Information:**

The online version contains supplementary material available at 10.1186/s42234-023-00140-3.

## Background

The gut-brain axis (GBA) describes the bidirectional communication between the gastrointestinal (GI) system and the central nervous system (CNS) (Pavlov and Tracey 2012; Sahasrabudhe et al. 2023; Breit et al. 2018). The vagus nerve is the primary neuronal branch of the gut-brain axis and contains afferent and efferent fibers. Vagal afferent neurons (VANs) relay chemosensory signals from the periphery to the CNS (Raybould and Zumpano 2021). The neuronal cell bodies of the VANs reside in the nodose ganglion, adjacent to the carotid artery. The VANs innervate the lining of the GI tract and express a variety of receptors capable of sensing chemical signals, such as bacterial metabolites and hormones, as well as viscerosensory signals, such as mechanical stretch (Li 2007; Egerod et al. 2018; Bai et al. 2019). Much of the knowledge on the GBA has been generated using in vivo models, where researchers aim to decouple the signaling routes from the periphery to the CNS. While in vivo studies provide the highest biological relevance, difficulty in controlling a large of number of experimental variables obfuscate the scientific process. In this case, in vitro models are better suited for isolating role of the VANs in sensing and relaying sensory information to the CNS.

Microfluidics-based approaches, broadly referred to as “organ-on-a-chip” or microphysiological systems, gained increased use for recapitulating anatomical layout of neurons, where neuronal processes and soma may reside in distal locations and hence exposed to different cues (Taylor et al. 2003). These platforms are generally fabricated using soft-lithography techniques involving molded polydimethylsiloxane (PDMS) bonded to glass slides, where two or more cell culture chambers are interconnected by an array of microchannels. In these platforms, neurons are seeded into one of the chambers and the neuronal process grow through the channels into the adjacent chamber. Microchannel dimensions are adjusted to restrict the cell bodies to one chamber and permit neuronal projections to extend into the opposing chamber (Goshi et al. 2022). These platforms have been used to model various physiological phenomena ranging from the culture of neural networks, studying the transport of aberrant proteins that play a role in neurodegeneration, investigating the spread of glutamate-induced excitotoxicity, as well as the innervation of non-neuronal cell cultures (Virlogeux et al. 2018; Peyrin et al. 2011; Iannielli et al. 2019; Polanco et al. 2018; Urrea et al. 2018; Brahic et al. 2016; Takeda et al. 2015; Song et al. 2014; Samson et al. 2016; Southam et al. 2013; Bellmann et al. 2019).

Here, we combine microfluidic approaches and vagal afferent neuron culture (Goshi et al. 2022; Girardi et al. 2023), to create a tool for studying the role of vagal afferent neurons in GBA while excluding the confounding signals from the circulatory and endocrine systems that are present in vivo. Specifically, we report on a microfluidic device that physically separates the VAN soma and neurite terminals to independently expose them to various stimuli, allowing us to probe the two independently. After studying the influence of microchannel geometry on neurite growth, we demonstrate VANs’ ability to uptake and directionally transport a verified retrograde tracer, cholera toxin-B (CTB), from the distal neurites to the soma. We show the electrophysiological response of the VANs upon separately exposing the soma and neurite terminals to mechanical and biochemical stimuli. Lastly, we confirm the presence of transient receptor potential cation channel subfamily V member 1 (TRPV1) receptors expressed along the neuronal processes, which indicates that the neurites express physiologically-relevant receptors at their distal projections.

## Methods

### Microfluidic device fabrication

Microfluidic devices were fabricated to separate VAN soma and neurite terminals. This was achieved by using a two-chamber device connected via microchannels. The chambers are 70 μm-high while the microchannels are 10 μm × 7 μm (w x h) at a length of 500 μm. The two-concentric chambers (*inside chamber* for cell bodies; *outside chamber* for neurite terminal) are connected with 32 radial microchannels.

The microfluidic devices were fabricated with standard soft lithography techniques. Briefly, master molds were fabricated by photolithographically patterning two layers of SU-8 (Kayaku Advanced Materials, MA, USA) on a silicon wafer. The first layer defined the microchannels and was fabricated using SU-8 6005 to achieve a thickness of 7 μm. The second layer defined the chambers and was fabricated using SU-8 2050 with a final thickness of 70 μm. Following the patterning of the chambers, the master mold was hard baked for 1 h at 250 °C. All dimensions of the microfluidic device master mold were confirmed using a Dektak XT profilometer (Bruker).

The master mold was placed into a 100 mm-diameter Petri dish and premixed (1:10 w/w curing agent to base) polydimethylsiloxane (PDMS, Sylgard 184, Dow Corning) was poured over the mold and placed into a vacuum chamber to remove any bubbles. The PDMS was then placed on a 90 °C hotplate for 3 h to cure. The microfluidic devices were peeled from the master mold and fluidic ports for cell seeding and culture media exchange were opened with 1.5 and 3 mm-diameter biopsy punches. Several microchannel designs (straight, open, angled) were fabricated to assess the influence of microchannel geometry on neurites extending from the inside chamber to the outside chamber.

Microfluidic devices were sterilized and irreversibly bonded prior to surface preparation for cell adhesion. Specifically, the PDMS devices and glass slides (Premium Cover Glass, Fisher Scientific, 12548B) were immersed in 70% ethanol, dried with N_2_ air, and placed into an air plasma cleaner at 10 W for 1 min (both sides). Microfluidic devices were then bonded to the glass inside a sterile biosafety cabinet and glass cloning cylinders (8 mm diameter, Sigma-Aldrich) were fixed over the two 3 mm ports via sterile vacuum grease to contain the culture media.

### Cell culture surface preparation

Glass slide surfaces enclosed by microfluidic devices were coated with poly-D-lysine (PDL, Gibco, A38904-01, 0.1 mg/ml) and Matrigel (Corning, 354230, 0.2 mg/ml, H_2_O) to promote cellular adhesion. Devices were incubated with PDL for 15 min, washed 5 times with sterile DI water, followed by incubation with Matrigel for 30 min, then rinsed and stored in incubator with sterile DPBS containing calcium and magnesium (DPBS + , Gibco, 14040133) until cell seeding. All solution additions were performed sequentially with the order of inside chamber, outer chamber (3 mm-diameter port), and then withdrawn from the outer chamber via 1.5 mm diameter port. Microfluidic device bonding and surface preparation was conducted within a few hours before cell harvesting.

### Nodose ganglia harvesting and culture

Male Wistar rats (5 weeks old) purchased from Envigo were used to obtain vagal afferent neurons. All tissue harvesting procedures were approved by the Institutional Animal Care and Use Committee at University of California, Davis. Animals were housed under 12 h light/12 h dark conditions and fed standard pellet chow ad libitum. Vagal afferent neurons were harvested following previously established protocols (Simasko et al. 2002). Briefly, nodose ganglia were isolated bilaterally from rats under anesthesia via isoflurane (VetEquip V-1 Tabletop with Active Scavenging). Following a midline incision in the neck, the musculature was retracted, and blunt dissection techniques were used to separate the vagal nerve from the carotid artery. Under magnification (AmScope LED-144A), each nodose ganglion was removed and placed in Hank’s balanced salt solution with calcium and magnesium (HBSS + 1X, Thermo Fisher, 14025092) while isolating the second ganglion. Once harvested, nodose ganglia were desheathed and digested in HBSS [-] (Gibco, 14185–052, H_2_O, 1x) with no calcium or magnesium, containing 1 mg/ml of both collagenase type 1A (Worthington Biochemical, LS004188) and dispase II (Roche, 04942078001) for 90 min at 37ºC in 95% air/5% CO_2_. The cells were dispersed via gentle trituration and then washed in cell culture medium (described next). Following two spin cycles of 200 g for 2 min, the pellet was resuspended in 50 µl of cell culture medium. The DPBS from the microfluidic devices was then removed from both chambers and 150 µl of fresh cell culture medium was added to the inside chamber. 25 µl of the cell suspension was then added to the bottom of the inside chamber for seeding. The devices were kept in the incubator for 1.5 h to allow for cell adhesion, after which point half the medium of the inside chamber was exchanged, while the outside chamber was filled with 150 µl of fresh cell culture medium. The devices were then returned to the incubator (37ºC in 95% air/5% CO_2_). Approximately half medium exchanges were performed (75 µl removed, 125 µl added) every 24 h to replenish the medium and account for evaporation until the experiments. The neurons required three days following seeding to grow through the microchannels and reach the outside chamber.

Cell culture medium is comprised of Dulbecco’s Modified Eagle Medium (DMEM, Thermo Fisher, 11885084, 46% v/v), Bovine Serum Albumin, (BSA, Sigma-Aldrich, A8806, DMEM, 0.5 mg/ml), GlutaMAX (Thermo Fisher, 35050061, 1.4 mM), Selenium/Insulin/Transferrin (ITS-G, Thermo Fisher, 41400045, 30 nM), Nerve Growth Factor (2.5S NGF, Envigo, B.5017, 125 ng/ml), and Ham’s F-12 Nutrient Mix (Thermo Fisher, 11765054, 49% v/v) (Ghogha et al. 2012). Medium contents are presented with the following format when appropriate: Name (Abbreviation, vendor, catalog number, solvent, working concentration).

### Immunocytochemistry and imaging

Cultures were washed 3 times with 37 °C DPBS + and fixed with 4% w/v paraformaldehyde (PFA, Affymetrix) in DPBS + for 1 h at room temperature. After fixation, the cells were washed three times with DPBS + , twice with 0.05% v/v Tween 20 (Sigma, DPBS +), followed by a 3-min permeabilization with 0.1% v/v Triton X-100 (Thermo Fisher, DPBS +) and two additional washes with the Tween 20 solution. The cultures were then blocked with 0.5% v/v heat-inactivated goat serum (Thermo Fisher) and 0.3 M glycine (Sigma-Aldrich) in DPBS + (blocking buffer) for 1 h. Following the blocking step, samples were incubated for 1 h with primary antibody solution containing mouse anti-βIII tubulin (βT-III; Thermo Fisher, 1:500) in blocking buffer (without glycine). For cultures stained for anti-transient receptor potential cation channel subfamily V (TRPV1), primary antibody solution contained mouse anti-βIII tubulin (βT-III; Thermo Fisher, 1:500) and rabbit anti-TRPV1 (Alomone Labs, 1:200) in blocking buffer (without glycine). Samples were then washed 3 times with Tween 20 solution followed by a 1-h incubation with secondary antibody solution containing either goat anti-mouse conjugated to AlexaFluor 555 (Thermo Fisher, 1:500) or both goat anti-mouse conjugated to AlexaFluor 555 (Thermo Fisher, 1:500) and goat anti-rabbit conjugated to AlexaFluor 488 (Thermo Fisher, 1:500) in DPBS + . Following incubation with the secondary antibody solution, the samples were washed 3 times with DPBS + , then incubated for 5 min with a 4’,6-diamidino-2-phenylindole (DAPI) solution (Sigma, 1:20,000, H_2_0). Samples were washed with Tween 20 a final time. Cultures on coverslips were mounted onto a glass slide (Globe Scientific, 1324B) using ProLong Gold Antifade Mountant (Thermo Fisher), while device cultures were filled with DPBS + and imaged immediately. Coverslip cultures were imaged using a Leica TCA SP8 STED 3X microscope with a 100x/1.4 oil immersion objective to visualize the presence of TRPV1 receptors. Devices were imaged using a Zeiss AX10 Observer.D1 with a 10 × and 20 × objective to visualize projections exiting the channels.

In order to quantify the influence of channel exit angle on neurite trajectory (i.e., tracking versus projecting), we pooled the data from four devices for neurites that completely exited the microchannels at the outside chamber. If a neurite remained adjacent to the channel wall upon exiting the channel, it was counted as “tracking”. Conversely, if a neurite departed from the channel wall and spread into the outside chamber, it was counted as “projecting”. The total number for each case was categorized with respect to the exit channel angle.

### Cholera toxin subunit B

Cholera toxin subunit B (CTB, Thermo Fisher, C34778, HBSS + , 10 µg/ml) was introduced into the outside chamber at day in vitro (DIV) 3 and devices were then incubated for 15 min at 37ºC in 95% air/5% CO_2_(Self et al. 2005). Following incubation, fluid in the outside chamber was aspirated, washed with fresh cell culture medium, and replaced with equal volume of medium. Devices were imaged with an epifluorescence microscope (Zeiss AX10 Observer.D1) around the outside chamber to visualize CTB uptake and obtain locations of the CTB in the projections. The same locations on the devices were then reimaged at time points of 2 and 24 h to assess spreading of CTB. Images were either collected with the same exposure time throughout all the time points or auto-exposed at each timepoint depending on the desired data (see Results section). To quantify the distance transported, the distance from channel entry to the leading edge of CTB signal was measured at the various time points. These quantities were then subtracted from one another to calculate the change in distance during a given time window. These values were then divided by the time allotted and averaged to calculate the average velocity of transport.

### Effector molecules

Capsaicin (Cap, Tocris, 0462, ethanol, 1 μM) was used to depolarize the neurons (Newberry et al. 2016; Riley et al. 2013) via the TRPV1 activation (Sasamura and Kuraishi 1999). Elevated extracellular potassium chloride (KCl, 20 mM) with an equimolar reduction of NaCl was used as a positive control (Rienecker et al. 2020). Ethanol (same working concentration with capsaicin) was used as the vehicle since other diluents (e.g., DPBS +) do not generally affect cell viability and function (Girardi et al. 2023).

### Intracellular calcium flux measurements

All calcium imaging was performed at room temperature (22 °C) and on DIV 4. Calcium transients were monitored by use of the fluorescent calcium indicator Fluo-4 AM (Thermo Fisher, F14201). Cells were prewashed with HBSS + , followed by incubation with 3 µM Fluo-4 AM for 60 min at room temperature (22ºC) in HBSS + , followed by another subsequent wash in HBSS + , per the protocol from the vendor. After the final wash, both chambers of the device were filled with 75 µl of HBSS + and placed on the microscope stage (Zeiss AX10 Observer.D1). The stimuli were introduced in the outside chamber in the following order: vehicle, capsaicin, and KCl. The samples were imaged around the wall of the inside chamber with a set exposure time of 400 ms after each stimuli. The neuronal cell bodies were identified by their large size (~ 30 μm diameter) and spherical shape, and marked as a region of interest (ROI) within ImageJ to extract their average intensity (Schneider et al. 2012). An inclusion criteria was used to remove dead cells from the analysis via the requirement of a neuron needing to exhibit a 10% increase over the baseline fluorescence when exposed to either Cap or KCl (Riley et al. 2013). All data from viable neurons were then pooled for each stimuli and relative fluorescence unit (RFU) changes across the groups were compared.

### Statistical analysis

An unpaired Student’s t-test with Welch correction was used to assess differences within projection lengths between straight and open channels. A one-way ANOVA with Tukey’s multiple comparison test was used to compare RFU of neuronal soma between various stimuli groups, as well as to compare exposure time of CTB loaded projections over time. For all statistical comparisons, a *p*-value < 0.05 was considered significant. All statistical analyses were performed in GraphPad Prism (version 10.0.2).

## Results

### Microfluidic device fabrication and VAN compartmentalization

**Fig. 1:**
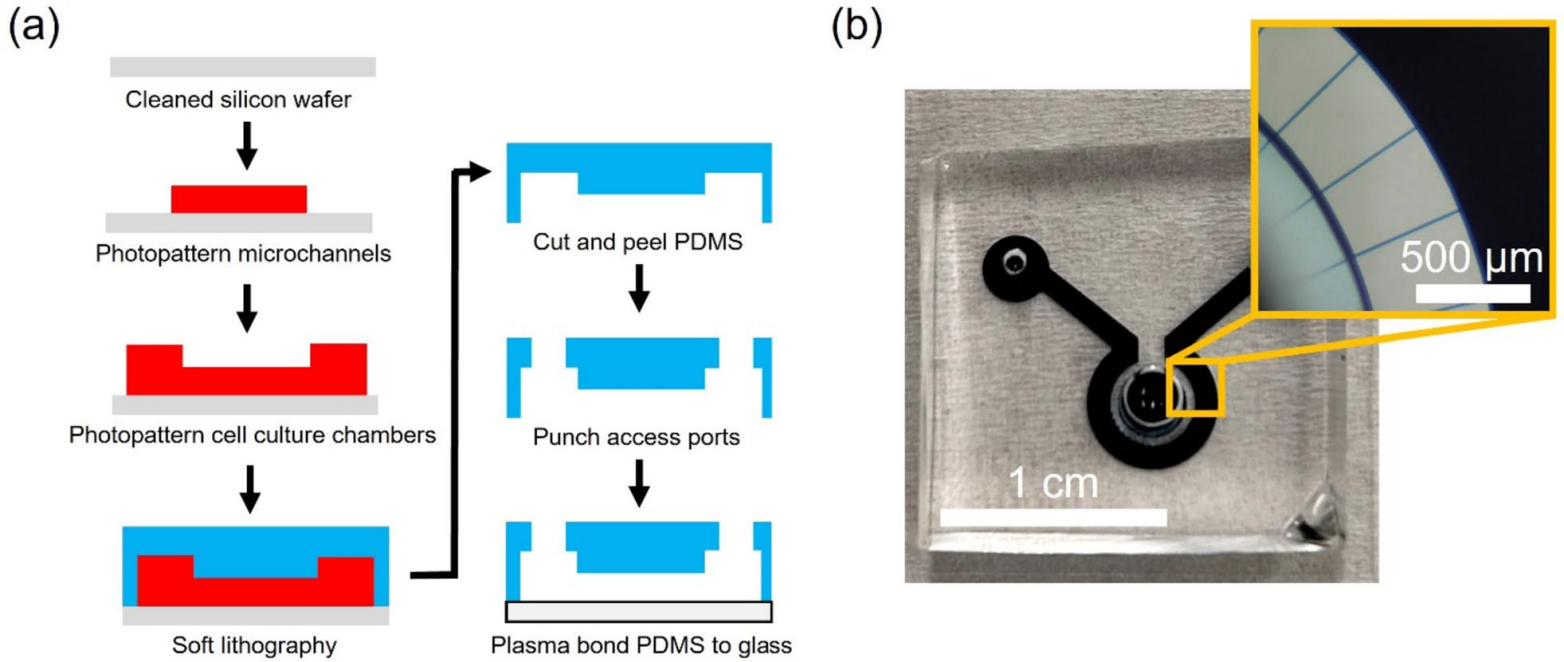
Microfluidic device fabrication. **a** Photolithography is used to create a negative mold of microfluidic devices, followed by soft lithography to transfer the mold pattern to PDMS and plasma-assisted bonding of PDMS to glass to create the device. **b** Microfluidic device filled with food coloring illustrates two concentric chambers connected via microchannels (inset)

Using photolithography techniques, a negative master mold for the microfluidic devices was fabricated. The master mold contained 14 copies of the microfluidic device, which consisted of 32 microchannels, 10 μm × 7 μm (w x h) and a length of 500 μm connecting two concentric chambers, height of 70 μm. Soft-lithography was used to create PDMS devices, which were then plasma bonded to glass to complete the microfluidic device (Fig. 1a). A device was filled with food coloring to visualize the two chambers and the interconnecting microchannels (Fig. 1b). Detailed device dimensions are shown in Fig. S1.

An adult rat contains 6,000 neurons per nodose ganglia, resulting in 12,000 neurons from a single rat (Cooper 1984). We studied different plating densities of these neurons and observed a relationship between density, cell migration, and projection growth. Neurons plated at 10 neurons per mm^2^ resulted in a sparse culture with large projections and minimal migration after 1 day in culture. Neurons plated at 700 neurons/mm^2^ led to neurons beginning to migrate towards one another and form clumps with minimal projections after 1 day in culture (Fig. S2a). We imaged these clumps and found that they are aggregates of neurons that eventually (~ DIV 4) grow networks of projections (Fig. S2b). Depending on the desired experiment, plating density can be varied to optimize cellular behavior. Moving forward for introducing nodose cultures into the microfluidic devices, we plated at around 350 neurons/mm^2^.

**Fig. 2:**
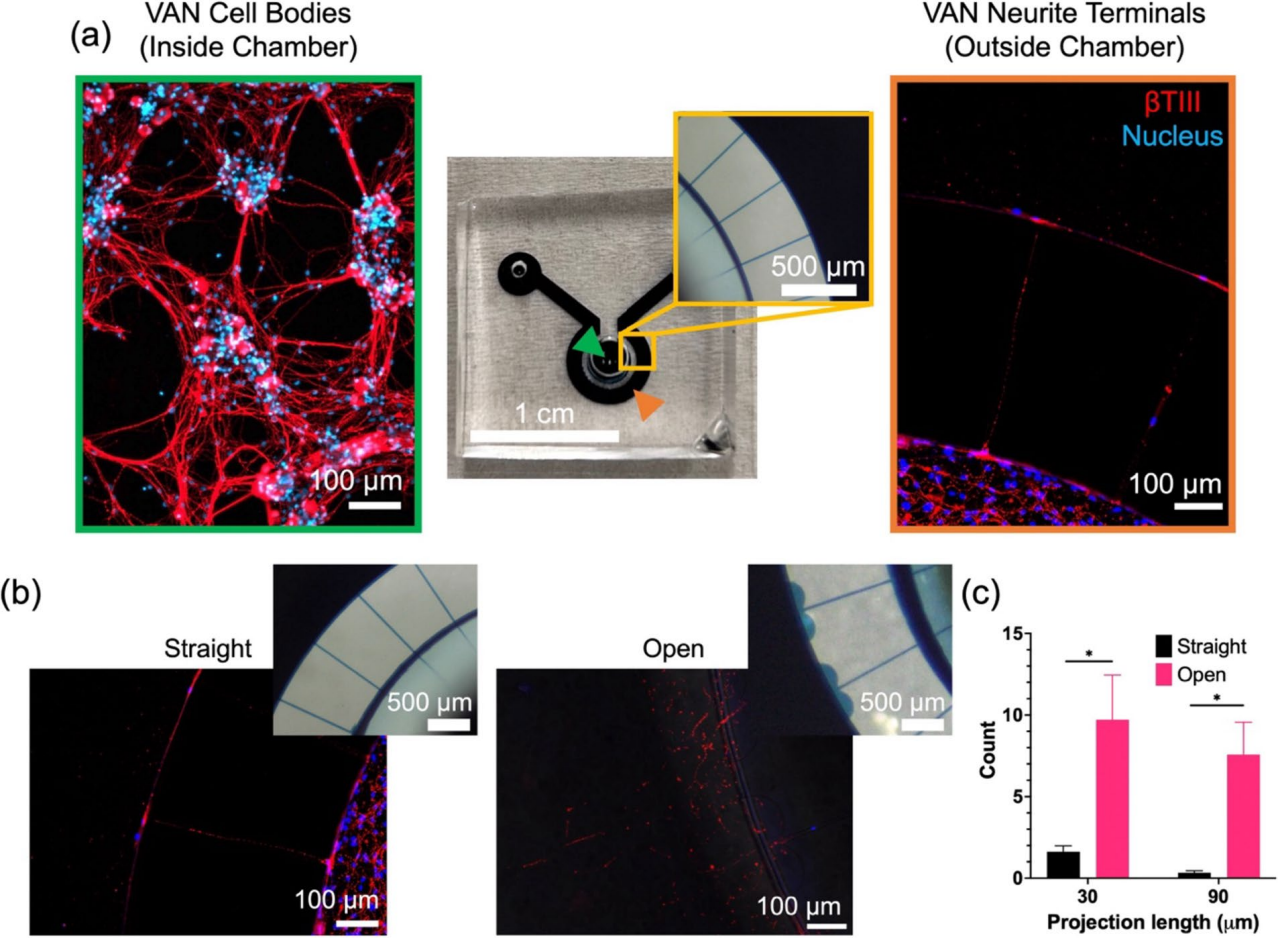
Primary vagal afferent neurons cultured inside microfluidic device (DIV 3). **a** Neurons are seeded into the inside chamber (green arrow), where neurites spontaneously grow through microchannels and reach the outside chamber (orange arrow). **b** Microchannel exit geometry (straight vs. open) influence neurite projection into the outside chamber. **c** Comparison of number of neurite projections that extend beyond 30 µm and 90 µm from the channel exit for straight (*n* = 27) and open (*n* = 7) channel exit geometries. * *p* < 0.05. Error bars indicate the standard error of the mean (SEM)

Dissociated nodose ganglion cells (including the VANs) were seeded into the inside chamber and allowed 3 days to grow neurites through the microchannels (Fig. 2a) for conducting the experiments (e.g., calcium imaging or transport studies) or immunostained for visualization. For immunostaining, neurons are stained for anti-βIII tubulin (shown in red) and DAPI (shown in blue). PDMS devices are irreversible bonded to the glass, so all staining occurs within the device, making it challenging to visualize projections inside microchannels. However, one can visualize projections entering and exiting the microchannels. Since the devices are fabricated with soft-lithography, a higher number of microchannels can be patterned by using custom photomask layouts to increase the probability of neurites traversing the microchannels. The cell bodies can be more successfully localized close to the microchannel entrances by taking advantage of hydrostatic pressure-driven convective flow from the inside chamber to the outside chamber, as demonstrated by others for CNS cells (Park et al. 2009).

### Microchannel exit geometry guides neurite projections

We observed that the microchannel exit geometry had a significant influence on whether neurites projected into the outside chamber or tracked along the chamber walls. Straight microchannel openings lead to neurites exiting the channels and tracking along the wall of the chamber, while open microchannel geometries lead to neurites exiting the channels and projecting into the outside chamber (Fig. 2b). Quantification of the projection distance into the outside chamber revealed that the open microchannels lead to significantly (*p < 0.05, unpaired t-test) more projections compared to straight microchannels (Fig. 2c).

**Fig. 3:**
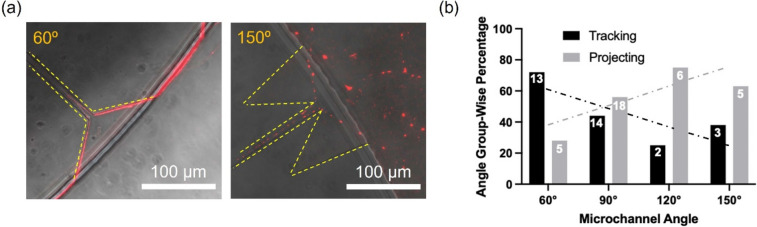
Microchannel exit angle guides neurites leaving the channels (DIV 3). **a** Representative images show two microchannel angle openings (60° and 150°), which dictate whether a neurite tracks along the chamber wall or projects into the chamber respectively. **b** Percentage of tracking and projecting neurites for neurite populations corresponding to each microchannel angle groups (60°, 90°, 120°, and 150°). The values within the columns indicate the number of neurites counted for each case. The least-squares-regression lines display the influence of microchannel angle on exiting neurite behavior

We systematically studied the effect of microchannel exit geometry on neurite guidance by varying the microchannel opening angle. The angle was varied from 60° to 150°, in steps of 30°. This was achieved via fabrication of a new mold, where the 32 microchannels were divided into 4 groups of 8 channels, with each group having one of these opening angles (Fig. S3). Again, the dissociated neurons were seeded into the inside chamber of the microfluidic device and immunostained after 3 days to visualize and evaluate the neurite projection behavior (Fig. 3a). Acute angles (e.g., 60°) resulted in a large fraction of neurites to track along chamber walls, whereas for obtuse angles (e.g., 150°) a larger number of neurites projected into the outside chamber (Fig. 3b).

### Cholera toxin subunit-B uptake and retrograde transport

**Fig. 4:**
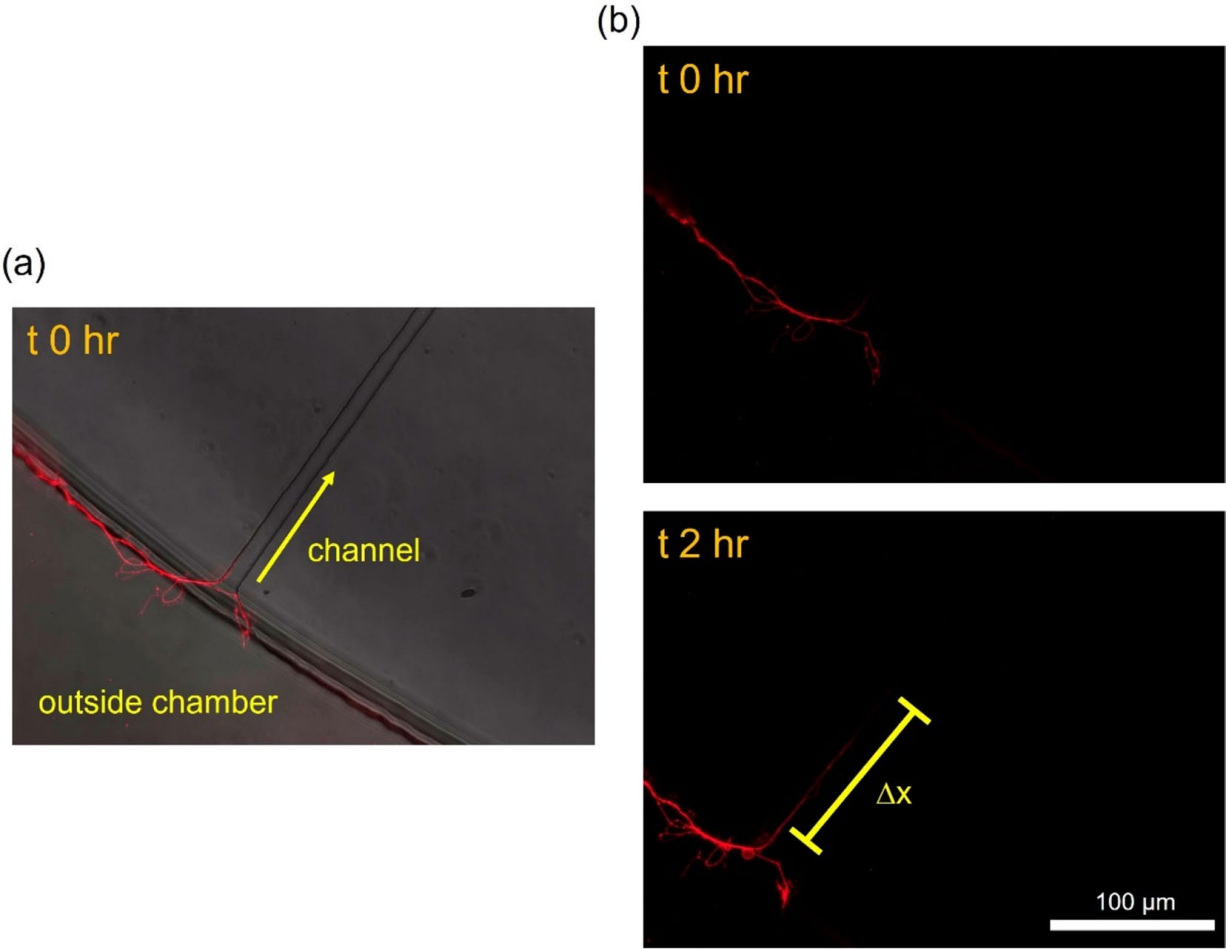
Cholera toxin subunit B (CTB) uptake and retrograde transport (DIV 3). **a** Fluorescently-tagged CTB is taken up at neurite terminals in the outside chamber. **b** CTB position at timepoint 0 and after 2 h, illustrating transport down the neurite through the channel. ∆x denotes the change in leading edge of transport position

When fluorescently-labeled CTB was introduced only to the outside chamber, which contains neurites exiting the channel, the neurites appeared to uptake the CTB proteins (Fig. 4a). Monitoring the same field-of-view over 2 h revealed that the fluorescence signal progressed along some neurites within the microchannels (Fig. 4b). To ensure that the observed retrograde transport of CTB is not due to its potential passive diffusion to the inside chamber via microchannels, we performed a control experiment using fluorescein, which has a molecular weight that is two orders of magnitude less than CTB, that is, it is a much faster diffuser than CTB. Briefly, fluorescein solution was introduced to the outside chamber, and the liquid heights were equalized to prevent convective transport. Within a 30-min duration (twice the incubation duration for CTB), there was negligible diffusive fluorescein transport to the inside chamber, as assessed by time-lapse fluorescence microscopy (Fig. S4).

**Fig. 5:**
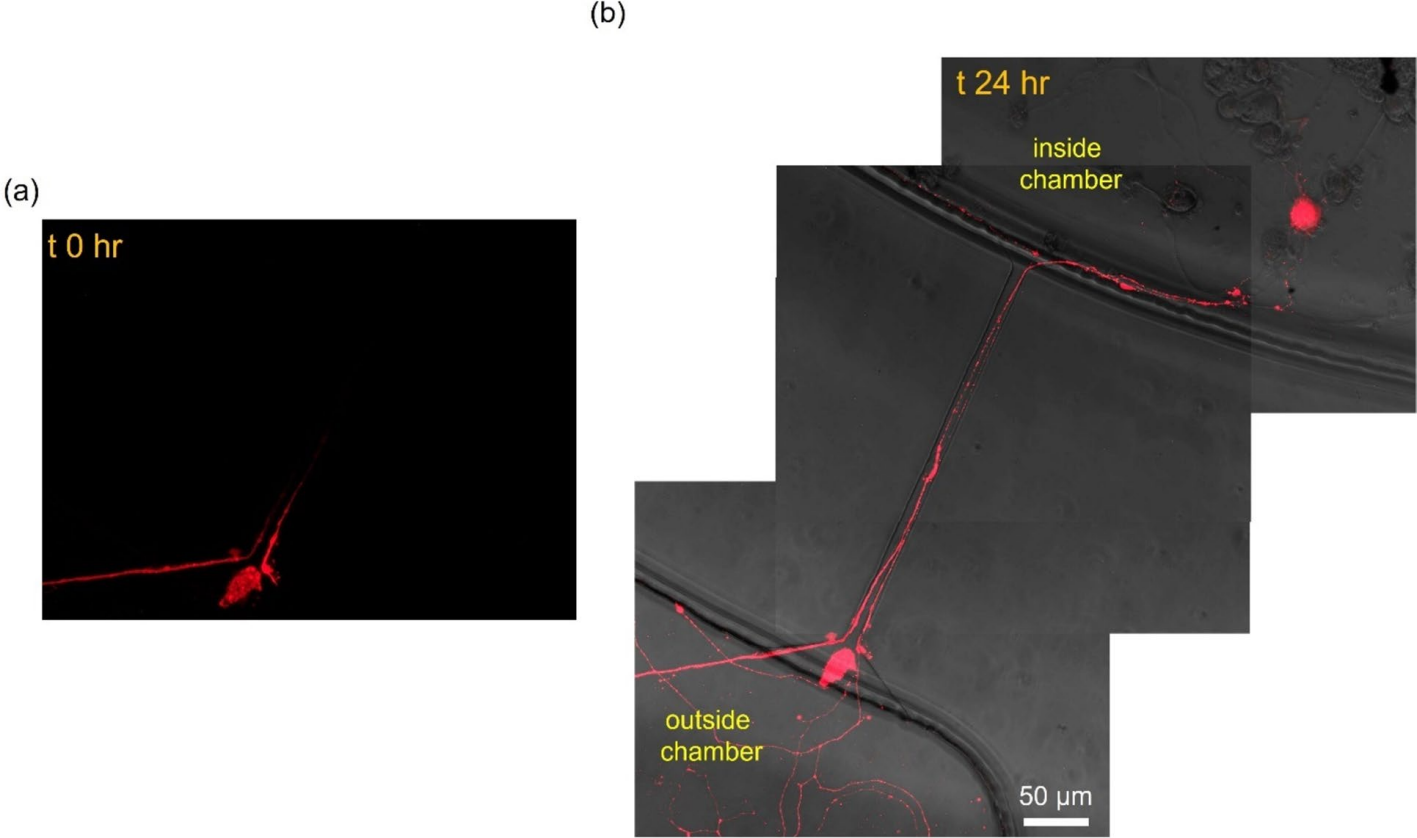
Cholera toxin subunit B (CTB) uptake and retrograde transport up to 24 h (DIV 3). (**a**) Uptake and retrograde transport at time 0 and 2 h. (**b**) At 24 h, CTB can be visualized inside the neuronal soma at the inside chamber

Through evaluation of immunostained samples pooled from 7 independent devices, we identified that 39 channels (out of 224 channels from the 7 devices) had a neurite projecting into the outside chamber. Of these 39 channels, 16 displayed CTB retrograde transport while the remaining 23 only exhibited protein uptake. We further analyzed these 16 cases and calculated transport velocity by measuring the distance the signal moved in unit time using ImageJ. The resulting transport velocity was 36.6 µm/hr or 0.6 µm/min. For one neurite, the fluorescence signal extended all the way back to the corresponding soma after 24 h (Fig. 5). Initially the signal was only localized at the nerve terminals upon CTB addition (Fig. 5a) but after 24 h it was present in the soma residing in the inside chamber (Fig. 5b). In addition, we imaged neurite terminals at the same field-of-view with auto-exposure at the various time points (0, 2, and 24 h), where the auto-exposure duration significantly increased after 24 h (Fig. S5).

### Mechanical and biochemical stimuli increase intracellular calcium activity

**Fig. 6:**
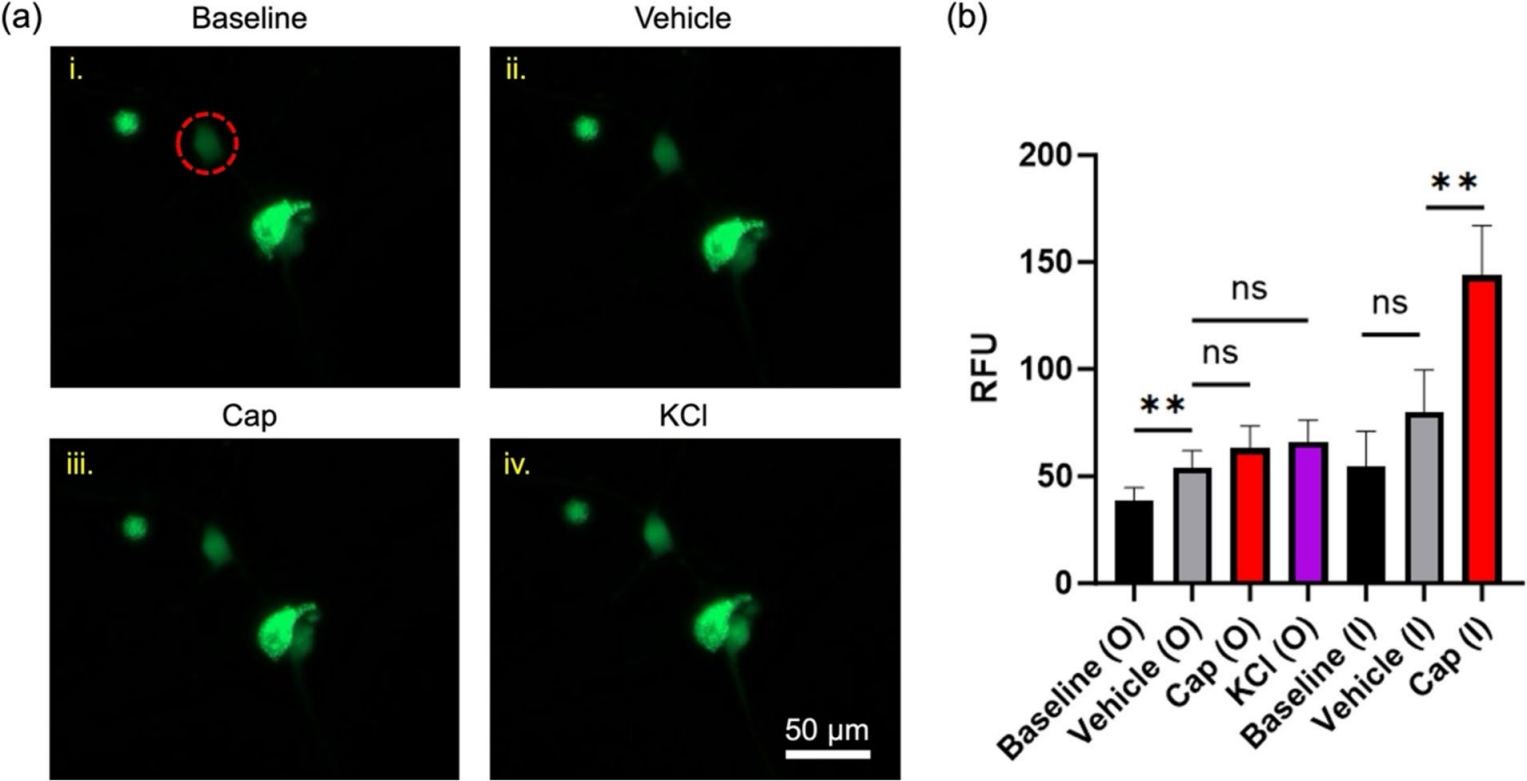
Intracellular calcium activity of neuronal soma at the inside chamber elevated in response to stimuli introduced at the outside chamber (DIV 4). **a** Neurons located at the inside chamber were sequentially exposed to (ii) vehicle, (iii) Cap (1 µM), and (iv) elevated extracellular KCl (20 mM) at neurite terminals. Red circle indicates a neuron whose intensity increases through the various exposure regimes. **b** Fluorescence intensity of neurons (RFU) compared between the various exposure regimes. O: stimuli introduced at outside chamber (*n* = 17 neurons), I: stimuli introduced at inside chamber (*n* = 11 neurons). ** *p* < 0.01. Error bars indicate the standard error of the mean (SEM)

Compartmentalization of the VAN cell body and neurite terminals also allows independently exposing either cellular region to external stimuli. Here, we sequentially introduced capsaicin (1 µM) then KCl (20 mM) to the outside chamber while imaging neuronal soma residing at the inside chamber. Fluorescence intensity due to intracellular calcium of a neuron (incubated with Fluo-4 AM calcium indicator) residing at the inside chamber increased through sequential additions of stimuli (Fig. 6a). Pooling data from viable neurons and comparing their mean intensity (RFU) revealed that fluorescence intensity at the soma significantly increased after vehicle solution addition to the outside chamber, while there was no significant increase in response to capsaicin or KCl compared to the vehicle (Fig. 6b). However, as a control, when the soma (instead of neurite terminal) was directly exposed to the same stimuli at the inside chamber, there was no significant increase from vehicle compared to the baseline; yet, there was a significant increase in fluorescence intensity for capsaicin exposure compared to the vehicle. Groups denoted with “O” represent stimulus at the outside chamber (*n* = 17 neurons) and the groups denoted with “I” represent stimulation at the inside chamber (*n* = 11 neurons).

### Capsaicin receptor expression on VAN soma and neurites

**Fig. 7:**
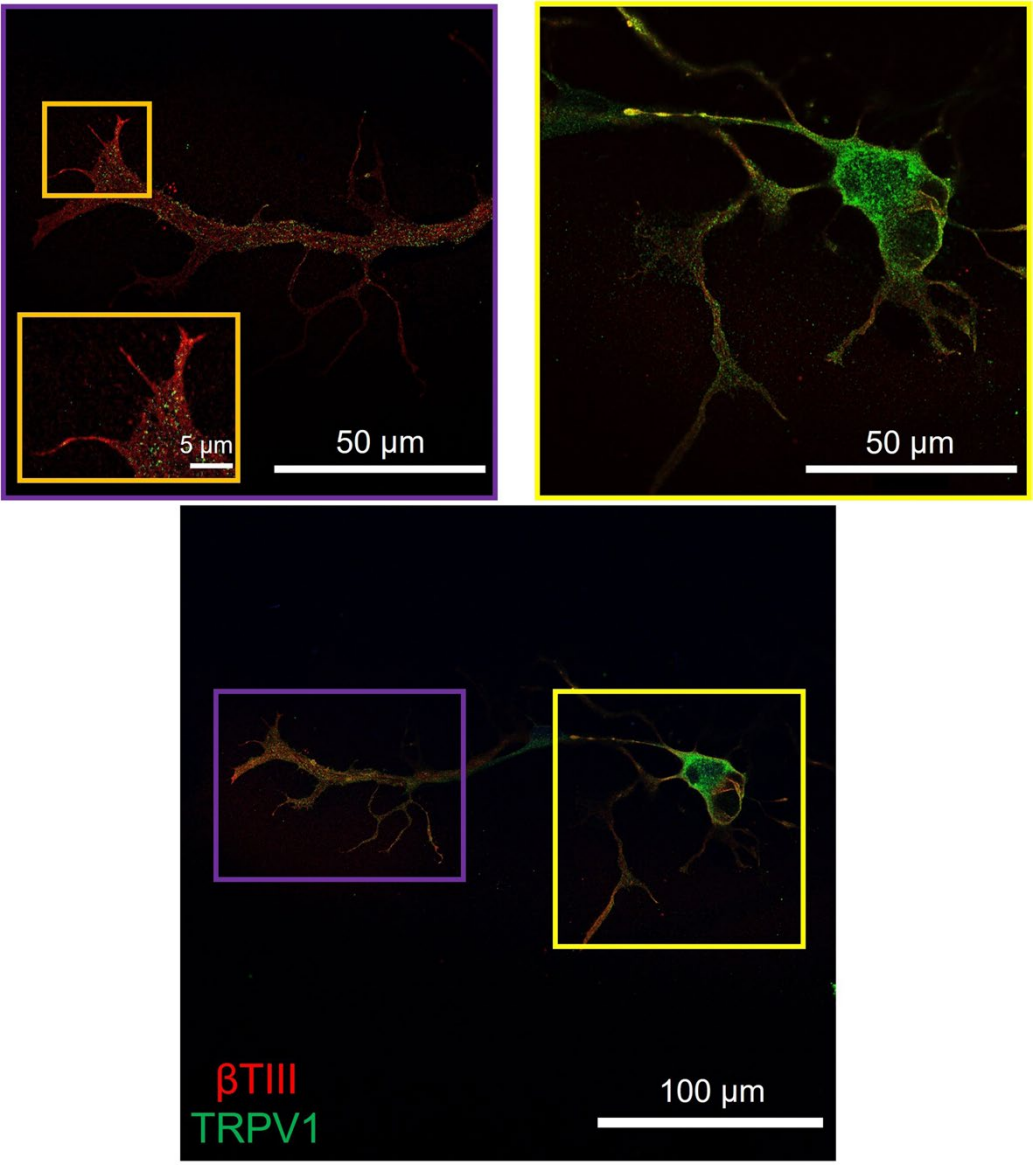
Immunostained capsaicin receptor (DIV 3). Anti-βIII-tubulin (red) displays overall neuronal morphology, while anti-TRPV1 (green) shows the presence of capsaicin receptors located both on VAN soma (yellow box) and neurites (purple box). Magnified view of neurite is shown in orange box inset

We immunostained VANs at DIV 3 for capsaicin receptor, TRPV1, across the entire neuron and imaged them via confocal microscopy to confirm the presence of receptors both at the soma (yellow box) and along the projections (purple box) (Fig. 7). As a negative control, we repeated the staining while omitting the primary anti-TRPV1 antibody, where there was no fluorescence signal (Fig. S6).

## Discussion

### Microfluidic compartmentalization of VANs

We have previously shown that acute primary VAN cultures exhibit elevated electrophysiological activity (confirmed by intra-cellular calcium activity and extracellular recordings) when exposed to physiologically-relevant gastrointestinal effector molecules, including capsaicin, serotonin, and cholecystokinin (Girardi et al. 2023). Here, we expanded on that work to demonstrate implementation of VAN cultures within microfluidic devices, where anatomically-relevant cases can be tested (e.g., retrograde transport or neurite terminal activation). Confounding physiological processes pose a significant challenge in studying the direct role of vagus nerve in the gut-brain axis in vivo. The in vitro model here aims to address this challenge by decoupling the contributions of the signals in the circulatory or endocrine systems and vagal signaling route to the central nervous system. This is achieved by utilizing microfluidic devices to model the anatomical layout of the vagal afferent neurons. Specifically, we used microfabrication techniques (i.e., photolithography and soft-lithography) to produce a microfluidic device conducive to dissociated VAN culture (Fig. 1). The device is composed of two chambers connected via microchannels. Once the dissociated VANs were seeded into the inside chamber, the neurite processes traversed the microchannels within three days and projected into the adjacent outside chamber. In this device, the microchannels restrict VAN somas to one chamber, but permit neurites to permeate the microchannels and enter into the adjacent chamber (Fig. 2). Interestingly, the microchannel geometry strongly influenced the behavior of neurites exiting the microchannels. Traditionally, microfluidic devices used for compartmentalizing cells contain interconnecting microchannels with rectangular outlets, where CNS neuronal projections readily sprout from the channels into adjacent chambers (Taylor et al. 2003). However, VAN projections tended to track along the chamber wall upon exiting channels. If the channel openings have an obtuse angle, the neurites transition from tracking along the walls to projecting into the outside chamber. We systematically explored this phenomenon by fabricating a device with various channel exit angles (60°, 90°, 120°, 150°) for culturing the VANs (Fig. 3). Neurites exiting channels with small exit angles (60°) had a higher percentage of tracking along the walls compared to their higher exit angle counterparts (120° and 150°), where these angles lead to a higher percentage of projecting. It is plausible that this phenomenon is due to neurons’ – at least peripheral sensory neurons – propensity to minimize free surfaces of their processes by either maximizing contact with a surface or another cell (Francisco et al. 2007). The latter is supported by the observation of VANs' tendency to form aggregates (Fig. S2). In order to carry this out, the VANs’ growth cones should be continuously sampling their path for mechanical and biochemical cues as they regenerate their processes. In the case of an acute angle of microchannel exit, is more probable for the growth cone to encounter a channel wall, hence, track along it (Fig. 3). Conversely, for obtuse angles, the abrupt discontinuity of the channel wall reduces the probability of the growth cone contacting the wall, hence, the neurite projects into the outside chamber. The ability to control neurite growth direction by modulating exit angle can also be broadly useful for controlling neuronal network formation.

We have demonstrated the ability to culture VANs multiple days (4 days) in contrast to prior studies mainly focusing on acute cultures with durations of 1 day or less (Simasko et al. 2002; Riley et al. 2013; Ragozzino et al. 2021; Troy et al. 2016). The extended culture duration allowed for VANs to develop sufficiently long neurites that can be incorporated into the microfluidic platforms for differentially compartmentalizing the soma and neurite terminals, mimicking the anatomical layout of the vagus nerve. This, in turn, created the capability to independently expose the soma and neurite terminals to different stimuli. Put another way, stimulating neurite terminals mimics direct signaling from the gut, while stimulating the soma mimics the influence of signals in the circulatory system on the cell body.

### Molecular transport and distal stimulation

Cholera toxin (CT) is a multimeric protein (AB5) secreted by the bacterium *Vibrio cholerae *(Vanden Broeck et al. 2007). CT binds to GM1 ganglioside that is commonly enriched on lipid rafts in the brain and it is subsequently endocytosed (Wands 2015). The B subunit of CT has been routinely used as a neuronal tracer due to its low toxicity. When the neurite terminals at the outside chamber were exposed to CTB, it was readily taken up by the VANs (Fig. 4), where 41% of the VANs exhibited retrograde transport of CTB (Fig. 5). The fluorescence localized at the neurites decreased over the course of day and higher exposure durations were necessary to register a fluorescence signal at the neurites (Fig. S5), suggesting that the internalized CTB diffuses throughout the cell. VANs exhibit significant cellular heterogeneity, which is observed as varying cellular functions such as differential electrophysiological response to distinct effector molecules (Girardi et al. 2023; Simasko et al. 2002). It is plausible that the heterogeneity also manifests itself in uptake and transport of proteins, including CTB, thereby resulting in a fraction of cells exhibiting transport of CTB, as also observed in in vivo tracing studies (Wang et al. 1998; Cailotto et al. 2012; Cui et al. 2022). Overall, the retrograde transport of CTB demonstrated here suggests that compartmentalized VANs can be used as a model to study the transport of other molecules, such as alpha-synuclein, a molecule which is hypothesized to migrate from the gut to the CNS resulting in neurodegeneration (Kim et al. 2019; Bindas et al. 2021).

In addition to transport studies, microfluidic VAN compartmentalization allows for studying electrophysiological response due to independent stimulation at the soma and neurite terminals. VANs readily exhibit increased intracellular calcium, captured by fluorescence microscopy, in response to capsaicin and elevated extracellular potassium chloride, as previously reported (Girardi et al. 2023; Riley et al. 2013; Rienecker et al. 2020). Here, we investigated whether isolated exposure on the neurite terminals to these molecules result in an increase in intracellular calcium activity at the soma. Switching the media from baseline to vehicle at the neurite terminal increased intracellular calcium at the soma (Fig. 6). The increased intracellular calcium due to the vehicle, which we reported previously (Girardi et al. 2023), can be explained by mechanical perturbation (removing/adding solution to the outside chamber), which mechanosensitive receptors expressed on VANs transduce the mechanical signal to action potentials that travel to the soma (Blackshaw et al. 2007). On the other hand, KCl exposure did not further increase the intracellular calcium level at the soma. KCl leads to nonspecific depolarization of neurons, although its concentration and exposure duration have been shown to result in contradictory neuronal responses (Rienecker et al. 2020), which may be a reason for no further increase in intracellular calcium due to KCl exposure of neurite terminals.

Exposure of the neurite terminals to capsaicin resulted in only a small increase in fluorescence signal in the cell body, which did not reach statistical significance. On the other hand, exposing the soma (residing in the inside chamber) to capsaicin resulted in a significantly elevated intracellular calcium compared to the response from neurite terminal exposure. Cap binds to TRPV1, a nonselective cationic channel that leads to an influx of Na^+^ and Ca^2+^(Sasamura and Kuraishi 1999), and elicits strong depolarization where its mechanism of action is well understood (Riley et al. 2013). We immunostained TRPV1 on VANs cultured on slides to assess TRPV1 expression on the soma and especially on the regenerating neurites (Fig. 7). There were high levels of TRPV1 expression at the soma and punctated presence along the neurites. Firstly, expression of TRPV1 is a promising indicator that the dissociated cells (neurites clipped around the soma for tissue harvesting) can successfully synthesize TRPV1 de novo in vitro and traffic the receptor proteins along the neurites. Even though spatial distribution of mechanosensitive receptors was not explicitly investigated here, the observed elevation in intracellular calcium at the soma upon mechanical perturbation at the neurite terminals suggest that these receptors (and possibly others) may also be synthesized and trafficked along the neurites. Secondly, lower density of TRPV1 expression at the neurite terminals may not be resulting in sufficient cationic influx to elicit action potentials that is necessary for increasing the intracellular calcium level at the soma. A more mature culture (> 4 days) may increase TRPV1 expression, and the VANs can be studied with patch-clamp approaches to reveal sub-threshold currents. Taken together, the studies here suggest that the VANs remain viable in these non-acute culture conditions and transduce stimuli (most potently due to mechanical perturbation) at the neurite terminals (outside chamber) to elevated intracellular calcium at the physically-separated soma (inside chamber).

## Conclusions

We have demonstrated a microfluidic device that physically separates VAN soma from their neurite terminals, thereby, more accurately recapitulating the anatomical layout of the vagal afferent neurons where the soma resides in the nodose ganglion and the neurite terminals are distributed across the peripheral organs. Systematic study of microchannel dimensions revealed that obtuse channel exit angles result in more of the neurites to project into the microfluidic chamber as opposed to tracking along the chamber wall. We demonstrated the proof-of-concept utility of the microfluidic platform in two physiologically-relevant cases. For the first example, we showed that CTB can be taken up by neurite terminals and retrogradely transported to the cell body. This observation can be expanded to study the transport of other molecules, including aberrant proteins originating in the gut that lead to CNS neurodegeneration or ingested environmental toxins. For the second example, we showed that the exposure of the neurite terminals to various stimuli, resulted in intracellular calcium elevation at the soma most significantly due to mechanical perturbations. In order to investigate the relatively small response to capsaicin exposure at the neurite terminal, we investigated TRPV1 distribution on the VANs. While TRPV1 was expressed on the newly grown neurites, their expression levels were not sufficient to elicit a significant increase in intracellular calcium at the soma. Future studies should investigate longer culture durations for obtaining a higher density of receptor proteins (including those other than TRPV1) and electrophysiological recordings with multiple electrode arrays and patch-clamp techniques. Overall, this work introduces a new tool to the gut-brain axis research community that should allow for controlled studies on decoupling various modes of gut-to-brain signaling (vagal versus circulatory or endocrine) and screening therapeutics.

### Supplementary Information

**Additional file 1.**

## Data Availability

The datasets generated during and/or analyzed during the current study are available from the corresponding author on reasonable request.

## Abbreviations

βTIII: β Tubulin III
ANOVA: Analysis of variance
BSA: Bovine serum albumin
Cap: Capsaicin
CNM2: Center for Nano/MicroManufacturing
CNS: Central nervous system
CT: Cholera toxin
CTB: Cholera toxin subunit B
DAPI: 4’,6-Diamidino-2-phenylindole
DI: Deionized
DMEM: Dulbecco’s modified eagle medium
DPBS: Dulbecco’s phosphate buffer solution
GBA: Gut-brain axis
H_2_O: Water
HBSS: Hank’s balanced salt solution
HBSS +: Hank’s balanced salt solution with calcium and magnesium
ITS: Selenium/Insulin/Transferrin
KCl: Potassium chloride
NGF: Nerve growth factor
PDL: Poly-D-lysine
PDMS: Polydimethylsiloxane
PFA: Paraformaldehyde
RFU: Relative fluorescence unit
ROI: Region of interest
TRPV1: Transient receptor potential cation channel subfamily V
VAN: Vagal afferent neuron
