# Supplementary material for: Microfluidic compartmentalization of rat vagal afferent neurons to model gut-brain axis
Source: Bioelectron Med. 2024 Feb 21;10:3. doi: 10.1186/s42234-023-00140-3 (PMC10880301; doi:10.1186/s42234-023-00140-3)
Supplement: Supplementary file 1 — Additional file 1. [file 42234_2023_140_MOESM1_ESM.pdf]

Supporting Information for

Microfluidic Compartmentalization of Rat Vagal Afferent Neurons to Model Gut-Brain Axis

Gregory Girardi[1], Danielle Zumpano[2], Helen Raybould[2], Erkin Seker[3]

Departments of [1]Biomedical Engineering, [2]Anatomy, Physiology, and Cell Biology, and [3]Electrical and Computer Engineering
University of California – Davis, Davis, CA 95616, USA

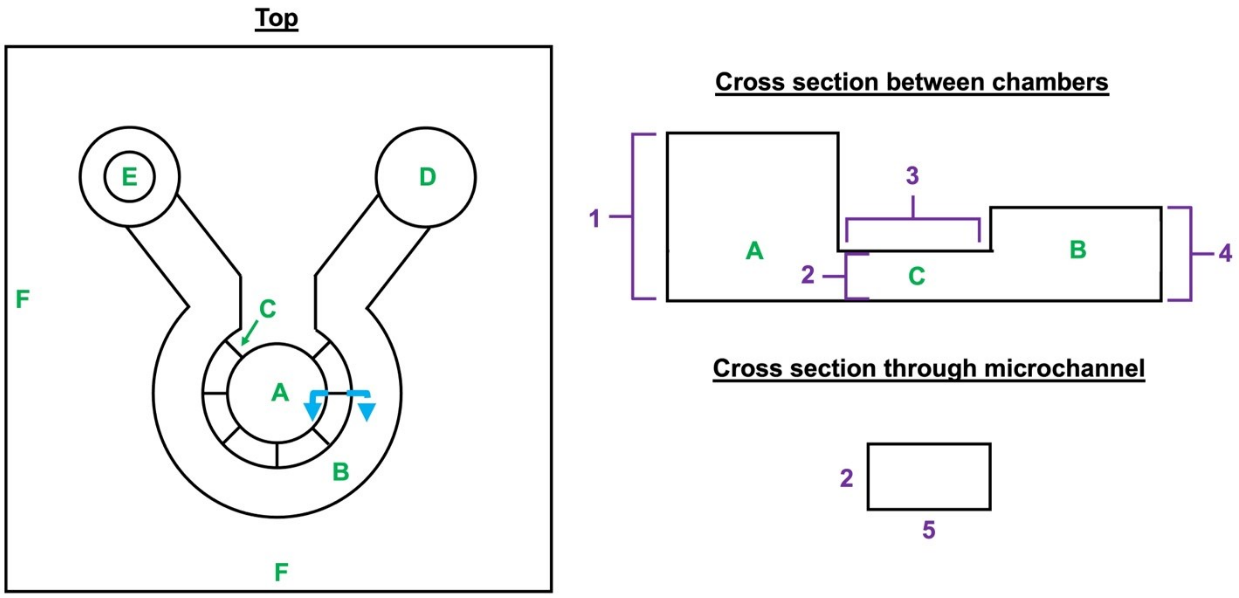

Figure S1. Microfluidic device dimensions. Top and cross-sectional views (shown with the blue arrow) of PDMS-based microfluidic device. **A** denotes the *inside chamber* that has a 3 mm diameter and has an open configuration to accommodate cell seeding and media exchange (1). **B** denotes the *outside chamber* that has a 70 μm height (4). **C** denotes the microchannels with 7 μm height (2), 500 μm length (3), and 10 μm width (5). **D** is the entry port to the outside chamber with a 3 mm diameter. **E** is the exit port to the outside chamber with a 1.5 mm diameter. **F** denotes 2.5 cm by 2.5 cm footprint of the entire microfluidic device.

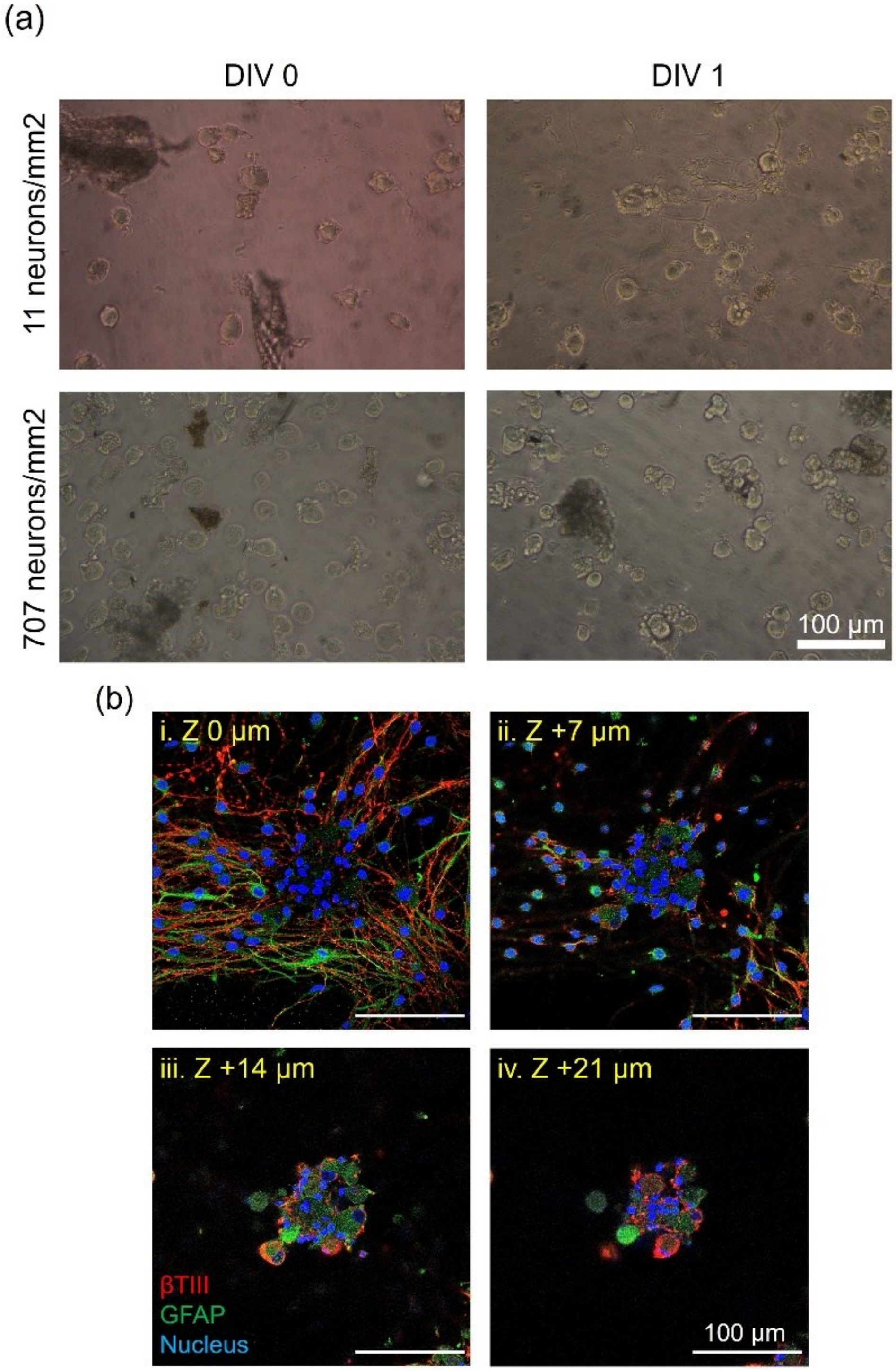

Figure S2. Role of plating density on vagal afferent neuron culture. (a) Neurons seeded at a low density of 11 neurons/mm^2 led to projections after 1 day *in vitro* (DIV) with negligible neuron clumping. Neurons seeded at a high density of 707 neurons/mm^2 led to no projections after DIV 1 while they migrated and began to form clumps. (b) A neuronal aggregate at DIV 4, which is immunostained for neurons (βTIII) and supporting cells (GFAP), reveals that neurons seeded at high densities form cell aggregates that eventually lead to projections, as shown at different z-planes (z=0 μm is the culture surface) of the aggregate (i – iv).

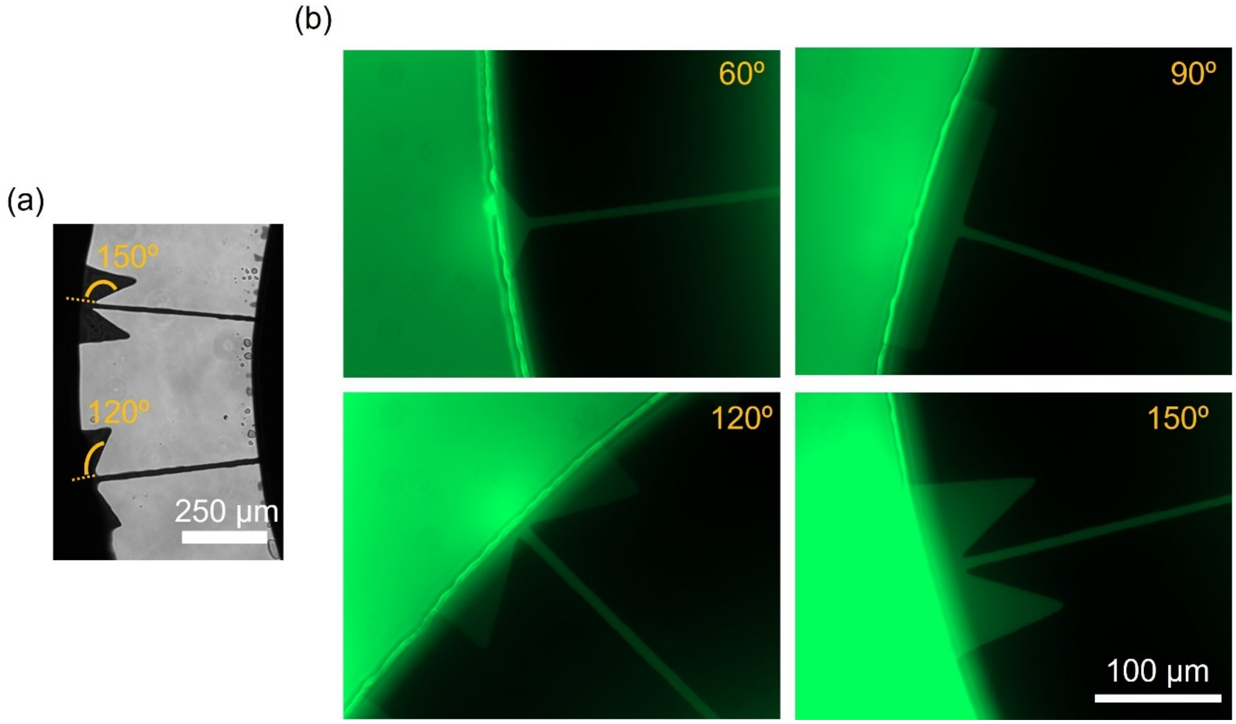

Figure S3. Microfluidic device with various exit channel opening angles. (a) Angle of opening represents the angle that the neuron encounters when exiting the channel. (b) Different channel exit geometries visualized via filling the device with fluorescein solution.

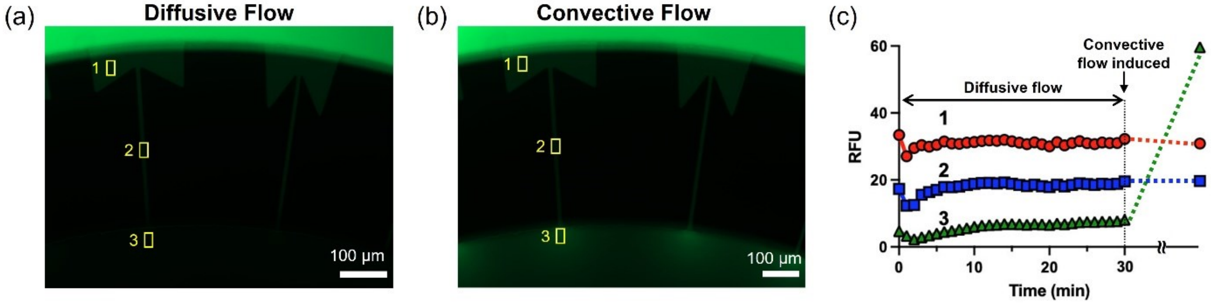

Figure S4. Assessment of fluorescein transport from the outside chamber to the inside chamber. (a) Fluorescein in HBSS was loaded in the outside chamber where the fluid height at inside and outside chambers were kept equal to prevent convective transport for 30 minutes. (b) At 30 minutes, the fluid height in the inside chamber was reduced, inducing convective transport. (c) The fluorescence intensity was monitored with an epifluorescence microscope during the experiment and quantified at three regions of interest (shown with yellow boxes). The plot shows negligible increase in fluorescence intensity at region-of-interest 3 (near the microchannel in the inside chamber), whereas upon induction of convective transport at 30-minute timepoint, there is an abrupt increase in fluorescence intensity due to fluorescein entering the inside chamber. Note that incubation with Alexa 488-labeled cholera toxin subunit B (CTB), which has two orders of magnitude higher molecular weight (hence exhibits slower diffusion) is 15 minutes (shorter than the duration of the test with fluorescein), suggesting that passive diffusion of CTB to the inside chamber housing the cell bodies should be negligible.

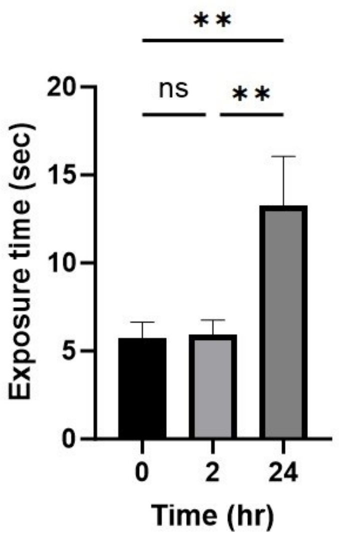

Figure S5. Image exposure duration at nerve terminals after CTB loading (DIV 3/4). (a) Exposure duration for imaging neurites exiting the channels at various time points: 0 hr (n = 9), 2 hr (n = 9), and 24 hr (n = 4) after CTB loading. Increased auto-exposure duration (i.e., dimmer neurites at 24 hours) suggests that CTB spread along the VAN. ** $p < 0.01$. Error bars indicate the standard error of the mean (SEM).

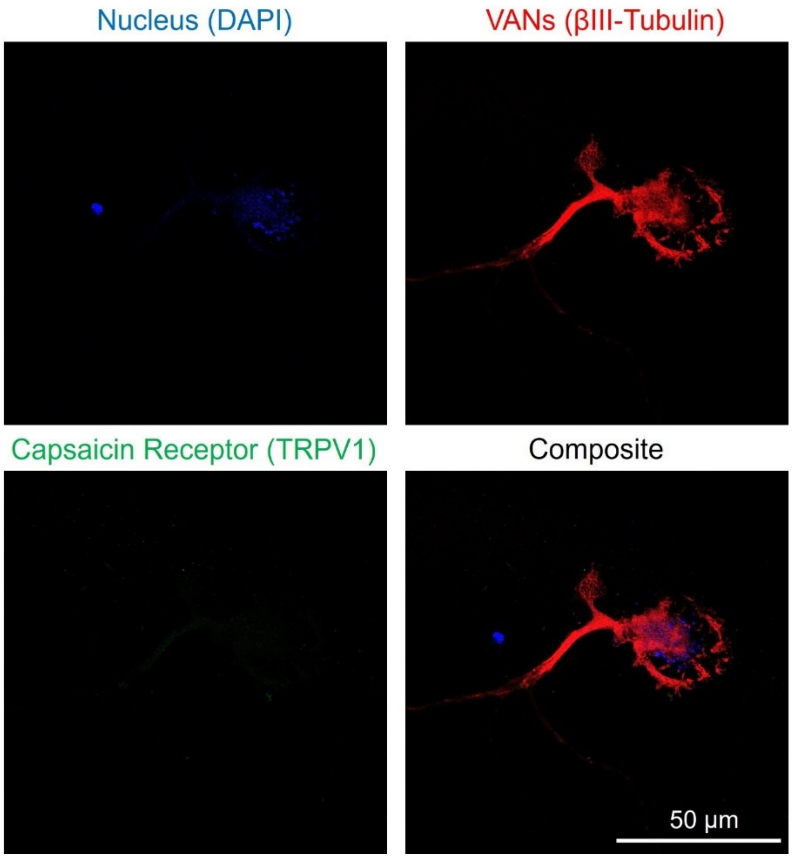

Figure S6. TRPV1 immunostaining negative control (DIV 3). Primary antibody (anti-TRPV1) was omitted during staining to evaluate non-specific staining by the fluorescently-tagged secondary antibody. When primary antibody was not used, there was no TRPV1 signal suggesting that the primary antibody specifically stains for TRPV1.
